# Natural Cotton Cellulose-Supported TiO_2_ Quantum Dots for the Highly Efficient Photocatalytic Degradation of Dyes

**DOI:** 10.3390/nano12183130

**Published:** 2022-09-09

**Authors:** Hancheng Shen, Weiwei Zhang, Chunyun Guo, Jing Zhu, Junjie Cui, Zhonghua Xue, Peirong Chen

**Affiliations:** Department of Applied Chemistry, School of Science, Anhui Agricultural University, Hefei 230036, China

**Keywords:** cotton cellulose, TiO_2_ quantum dots, photocatalysis, degradation, dyes

## Abstract

The artificial photocatalytic degradation of organic pollutants has emerged as a promising approach to purifying the water environment. The core issue of this ongoing research is to construct efficient but easily recyclable photocatalysts without quadratic harm. Here, we report an eco-friendly photocatalyst with in situ generated TiO_2_ quantum dots (TQDs) on natural cotton cellulose (CC) by a simple one-step hydrothermal method. The porous fine structure and abundant hydroxyl groups control the shape growth and improve the stability of nanoparticles, making natural CC suitable for TQDs. The TQDs/CC photocatalyst was synthesized without the chemical modification of the TQDs. FE-SEM and TEM results showed that 5–6 nm TQDs are uniformly decorated on the CC surface. The long-term stability in photocatalytic activity and structure of more than ten cycles directly demonstrates the stability of CC on TQDs. With larger CC sizes, TQDs are easier to recycle. The TQDs/CC photocatalysts show impressive potential in the photocatalytic degradation of anionic methyl orange (MO) dyes and cationic rhodamine B (RhB) dyes.

## 1. Introduction

Over the past few decades, the rapid development of modern industries worldwide has led to a severe cascade of problems in the water environment and human health [1,2,3]. Organic dyes, such as rhodamine B (RhB) and methyl orange (MO), are bulk and commonly used chemicals in the textile and apparel industries that are lost in a large portion (~20%) during the synthesis and manufacturing processes and are discharged into the water [4,5]. These colored dyes in wastewaters are poisonous and detrimental to human beings and prove challenging to degrade due to their high structural stability and low biodegradability [6,7,8]. Therefore, removing the dyes from wastewater is substantially vital for the development of a more environmentally sustainable earth. To this end, various technologies have been developed, including biological degradation [9,10], flocculation [11], adsorption [12,13], advanced oxidation processes (AOPs) [14], and membrane separation [15], which somewhat suffer from inert kinetics, complicated processes, high cost, and low efficiency.

Photocatalytic oxidation is one of the AOPs that has great potential to completely degrade a wide range of organic dyes by using solar energy [16,17]. To date, TiO_2_ has been widely used as an inexpensive and efficient semiconducting photocatalyst for the oxidative degradation of dyes, attributing to its ideal properties such as wide tunable bandgap (~3.2 eV), high stability, and ease of availability; however, its photocatalytic activity is still plagued by the sluggish separation of charge carriers and limited surface-active centers [18,19,20]. In this regard, the shape controlling of TiO_2_ crystals into a nano-size can elevate the photocatalytic capacity by affording more active sites from the unit mass [21]. For example, TiO_2_ quantum dots (TQDs) generally show enhanced photocatalytic activity compared to bulk TiO_2_ because of both size effect and optimized band structures [22,23]. Nonetheless, TQDs are rather prone to agglomerate and lack sufficient reusability, thus reducing their final photocatalytic performance and real-world application value [24]. On the other hand, it has become a new trend to use recycled materials to support photocatalyst powders. As a result, an effective approach to developing supported TQDs that govern their activity is highly needed.

Cotton cellulose (CC) is the most important renewable biopolymer used in a range of applications from the textile industry to medical, accounting for about 35% of the world’s total annual cellulose demands [25,26]. CC is an ideal support for photocatalysts because of its superfine porous structure and rich hydroxyl groups [27]. The superfine structure and rich hydroxyl groups not only provide support but also help to control particle shape growth and enhance particle stability by providing a template surface [28,29]. Yu et al. reported a one-pot synthesis route to fabricate CNC/ZnO nanohybrids, which homogeneously dispersed ZnO nanoparticles anchored onto the cellulose nanocrystal (CNC) surface [30]. ZnO nanoparticles were uniformly distributed on CNC with an average diameter of 42.6 nm. Furthermore, using CC as a supporting photocatalyst overcomes the shortcomings of difficulty in the collection and removal of photocatalysts in water. Using micron-size CC as the carrier for TQDs makes the recycling of TQDs more simply.

Herein, we present the preparation of TQDs grown in situ on CC biopolymer as an efficient and stable photocatalyst (TQDs/CC) for the photodegradation of organic dyes. The CC serves as a natural and green biopolymer support to stabilize the TQDs, triggering increased activity and significantly improved photocatalyst reusability [31,32]. FE-SEM and TEM confirm the formation of 5–6 nm TQDs on the CC surface. The photocatalytic activity of TQDs/CC remained unchanged after ten cyclic photocatalytic degradation tests, and EDX mapping shows that TQDs still decorated on the CC surface. The results demonstrate that TQDs/CC has great application potential in the photocatalytic rapid decomposition of different types of dyes, such as anionic MO dyes and cationic RhB dyes.

## 2. Experimental Section

### 2.1. Materials

Cotton cellulose (CC) was obtained from Haishihainuo Bosswin Medical Treatment Articles Co., Ltd. (Qingdao, China). Tetrabutyl titanate (TBOT, 98%) was purchased from Tianjin Kermel Reagent Co., Ltd. (Tianjin, China). Nitric acid (HNO_3_, 65–68%) and anhydrous ethanol (C_2_H_5_OH, 99.7%) were purchased from Sigma-Aldrich (Tianjin, China). Methyl orange (MO) and rhodamine B (RhB) were acquired from Shanghai Macklin Biochemical Co., Ltd. (Shanghai, China). All reagents and materials were directly used without further purification. Deionized water was used in all experiments.

### 2.2. Preparation of TQDs/CC Photocatalyst

TQDs/CC photocatalyst was synthesized by a hydrothermal method. Typically, 0.5 g clean CC, 1 mL HNO_3_, and 15 mL deionized water were mixed and stirred thoroughly to obtain solution A. Solution B was 15 mL ethanol containing a certain amount of TBOT. Solutions A and B were mixed and stirred for 20 min to obtain the precursor solution. Subsequently, the precursor solution was heated at 180 °C for 10 h in a Teflon-lined autoclave. After cooling naturally, the product was washed with ethanol several times and then dried to obtain TQDs/CC-*x* samples, where “*x*” represents the grams of TBOT.

### 2.3. Characterization

Field emission scanning electron microscopy (FE-SEM) and corresponding energy-dispersive X-ray spectroscopy (EDX) images were taken from a Hitachi S-4800 scanning electron microscope (Hitachi Inc., Tokyo, Japan). Transmission electron microscopy (TEM), high-resolution TEM (HR-TEM) observations and selected area electron diffraction patterns (SAED) were performed over an FEI Tecnai F20 microscope (FEI Inc., Hillsboro, OR, USA). Fourier transform infrared (FT-IR) spectra were recorded by a Nicolette IS50 instrument (Thermo Fisher Scientific Inc., Waltham, MA, USA). X-ray diffraction (XRD) patterns were taken on a Bruker D8 Advance X-ray diffractometer with Cu Kα radiation (λ = 1.5418 Å) (Bruker Inc., Karlsruhe, Germany). X-ray photoelectron spectroscopy (XPS) results were obtained using a Thermo Scientific k-alpha spectrometer (Thermo Fisher Scientific Inc., Waltham, MA, USA) with an operating voltage of 12 kV and Al Kα X-ray radiation (*hʋ* = 1486.6 eV). Then, UV-vis diffuse reflectance spectra (DRS) were used to study the optical properties of the samples with a Shimadzu UV-3600i Plus UV-vis spectrophotometer (Shimadzu Inc., Kyoto, Japan), using BaSO_4_ as a reflectance standard.

### 2.4. Photocatalytic Measurements

MO and RhB are typical anionic and cationic dyes, respectively. The 10 mg/L MO or RhB in water was used as a simulated dye pollutant solution. The photocatalytic performances of the samples were performed under UV light of 254 nm irradiated by a 30 W low-pressure mercury lamp. Typically, 50 mg of the dried samples were added to 25 mL of MO or RhB solution. The suspension was stirred in the dark or under UV irradiation for 0.5 h to realize adsorption-desorption equilibrium. Afterward, 5 mL of suspension was taken out every 0.5 h and centrifuged for 3 min. The absorbance of MO and RhB dye were measured under 465 and 554 nm, respectively. The photodegradation efficiency of the catalyst was calculated by using the following equation: *X*% = *C*/*C*_0_ × 100%, where *X*% is the photodegradation efficiency, *C*_0_ is the initial concentration of the dye, and *C* is the concentration of dye after the photocatalytic experiment.

## 3. Results and Discussion

### 3.1. Preparation of TQDs/CC Photocatalyst

**Figure 1:**
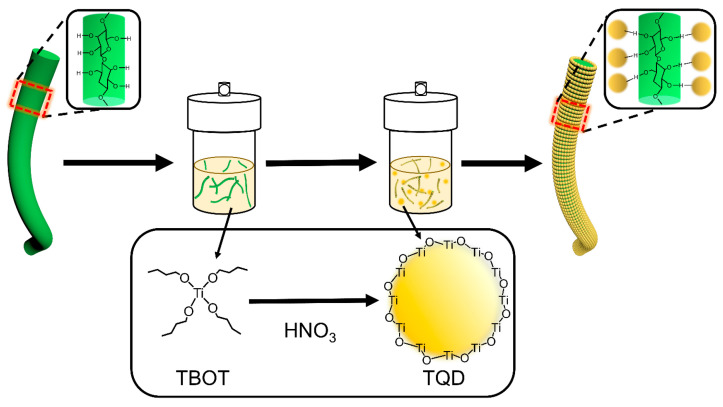
Synthetic process of TQDs/CC photocatalyst.

Figure 1 shows the *in-situ* synthesis of the TQDs/CC photocatalyst by directly depositing TQDs onto the natural CC via a hydrothermal process. In detail, TBOT was hydrolyzed in ethanol to generate titanium hydroxide (real reaction process: Ti(C_4_H_9_O)_4_ + 4H_2_O → Ti(OH)_4_ + 4C_4_H_9_OH), which decomposed into TQDs with the existence of HNO_3_ under high temperature and pressure (Ti(OH)_4_ → TiO_2_⋅nH_2_O → TQDs) [33,34]. The as-formed TQDs were chemically absorbed with the hydroxyl group of CC by hydrogen bonding and were further stacked layer-by-layer through the van der Waals forces, thus assembling a thick layer of TQDs on the surface of CC [28,35]. It has been reported that TiO_2_ is coated with amino groups and then adheres to cotton fibers through hydrogen bonding under heat treatment [36]. Our work, in contrast, deposited TiO_2_ quantum dots directly onto cotton cellulose through hydrogen bonding or van der Waals forces without TiO_2_ chemical modification in a one-step hydrothermal method.

### 3.2. Characterizations of TQDs/CC Photocatalyst

**Figure 2:**
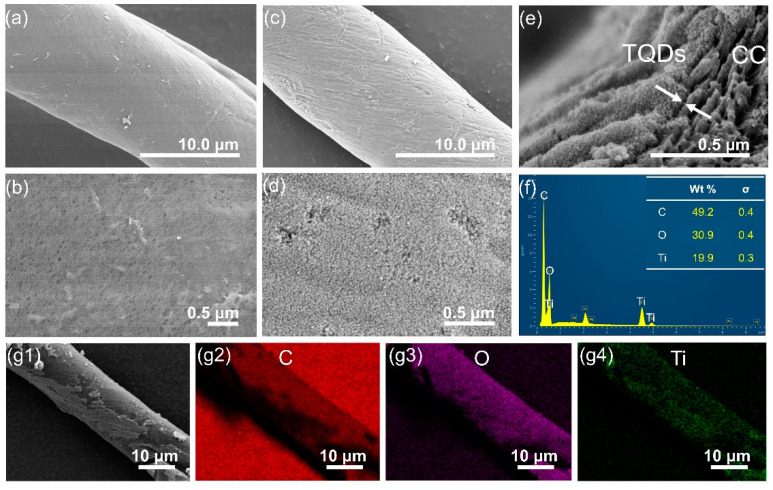
(**a**,**b**) SEM images of CC. (**c**–**e**) SEM images and corresponding elemental analysis (**f**) and SEM-EDX mapping results after an ultrasonic treatment (**g1**–**g4**) of the TQDs/CC-2.5 sample.

The morphology and structural characteristics of CC support and TQDs/CC photocatalyst were investigated by FE-SEM. Figure S1 presents that TQDs/CC photocatalyst, like CC, consists of fibers with several hundred micrometers in length and an average diameter of about 12–13 μm. From FE-SEM images with higher magnification, it can be found that the surface of CC is relatively smooth (Figure 2a), and there are many holes in CC itself (Figure 2b). The surface of CC changes from smooth to rough, coated with dense nanoparticles (Figure 2c,d) after the hydrothermal process, and the holes are no longer observed, indicating the successful deposition of TQDs onto the surface of CC. A clear interface between TQDs and CC support can be observed in Figure 2e. As illustrated in Figure 2e, the porous fine structure of CC provides conditions for the growth of the TQDs layer. The corresponding elemental analysis by SEM-EDX (Figure 2f) further verifies that C, O, and Ti are the main constituent elements for the TQDs/CC sample. The sample was dispersed in water and sonicated for 2 h by using an ultrasound cleaning bath. The effect of ultrasound treatment is due to acoustic cavitation, which provides unique physical effects based on mechanical effects such as shear forces, microjets, and shock waves [37]. Thus, these extreme reaction conditions would result in the exfoliation of TQDs from CC. Furthermore, SEM-EDX mapping results (Figure 2(g1–g4)) indicate substantial quantities of residues of TQDs on the CC surface after an ultrasonic treatment for more than 2 h, unambiguously demonstrating the high chemical and physical stability of the TQDs/CC photocatalyst.

**Figure 3:**
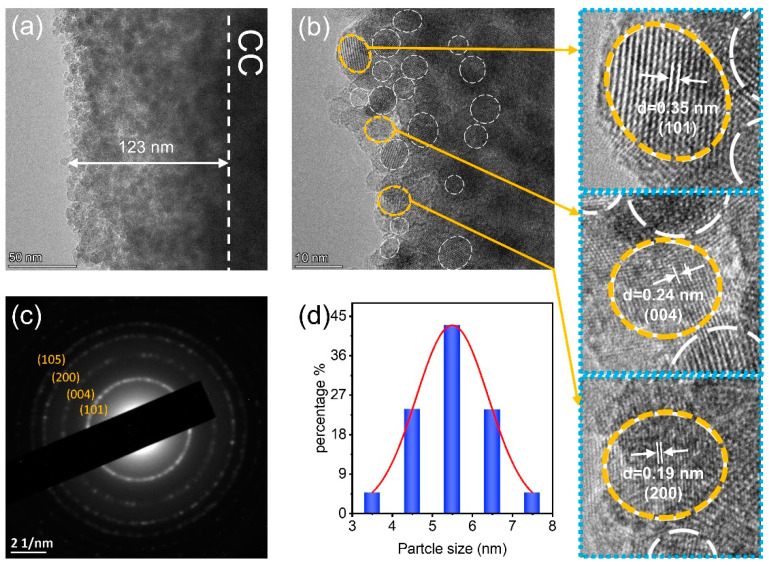
TEM (**a**), HR-TEM (**b**), SAED (**c**) images, and (**d**) particle size distribution of TQDs/CC sample.

To investigate the more detailed structure of the TQDs/CC photocatalyst, the TEM images are shown in Figure 3. Figure 3a reveals that the thickness of the TQDs layer is about 123 nm, and TQDs are firmly attached to the CC. Figure 3b and d display that the particle size of TQDs ranged from 3 to 8 nm. The crystal lattice fringes of circled TQDs in Figure 3b with a spacing of 0.35, 0.24, and 0.19 nm correspond to the (101), (004), and (200) planes of anatase TiO_2_, respectively [38]. The SAED of the TQDs/CC photocatalyst (Figure 3c), same as HR-TEM, shows the sharp diffraction rings of anatase TiO_2_, in which the diffraction rings are attributed to (101), (004), (200), and (105) of anatase TiO_2_, respectively [39,40]. No diffraction ring of CC appears in the SAED of TQDs/CC photocatalyst, which proves that the CC surface is covered with a thick layer of TQDs.

**Figure 4:**
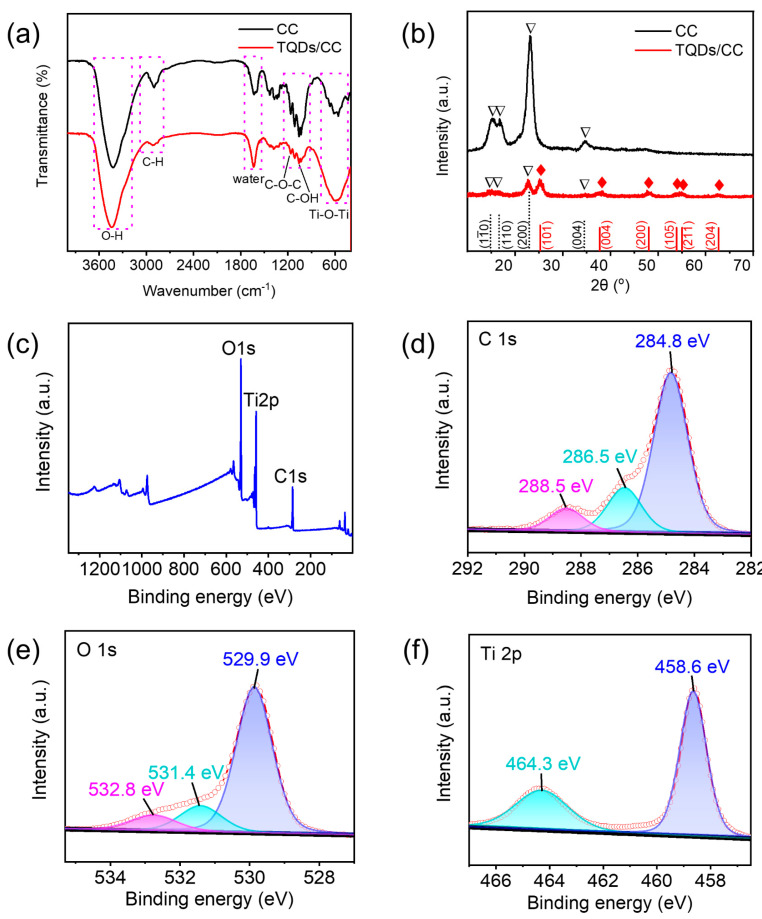
(**a**) FT-IR spectra and (**b**) XRD patterns of CC and TQDs/CC samples (Triangle represents peaks of cellulose and rhombus represents peaks of anatase TiO_2_). XPS survey (**c**), high-resolution C 1s (**d**), O 1s (**e**), and Ti 2p (**f**) XPS spectra of TQDs/CC.

The FT-IR spectra of CC and the TQDs/CC photocatalyst are shown in Figure 4a. Broad bands of 3000–3800 cm^−1^ are assigned to the stretching vibration of the O-H bond of the hydroxyl, while the characteristic peak observed at 2904 cm^−1^ shows C-H in the cotton structure [41]. In the FT-IR spectra, the peaks of 1166 and 1063 cm^−1^ correspond to C-O-C and C-OH, respectively. The band at 1634 cm^−1^ is related to the bending mode of the water [42]. Furthermore, compared with CC, the C-H, C-O-C, and C-OH, the intensity peaks of the TQDs/CC photocatalyst were significantly weakened, indicating the presence of TQDs on the surface of CC. Meanwhile, FT-IR broad bands of TQDs/CC photocatalyst at 500–800 cm^−1^, corresponding to the stretching vibration of the Ti-O-Ti (Figure S2), suggests the possibility of interactions between the TQDs and CC [43,44]. This result indicates the successful formation of the TQDs/CC photocatalyst.

CC and TQDs/CC photocatalyst crystal phases were analyzed by XRD. As shown in Figure 4b. The XRD pattern of CC, same as the TQDs/CC photocatalyst, shows four typical peaks at 2*θ* = 14.9°, 16.5°, 22.6°, and 34.3° corresponding to the diffraction planes (1$\bar{1}$0), (110), (200), and (004) of cellulose Iβ, respectively (JSPDS No. 56-1718) [45,46]. However, the diffraction peak intensity of (200) matched to CC in the TQDs/CC photocatalyst decreases sharply due to the existence of TQDs. The XRD pattern shows the diffraction peaks at 25.4°, 37.9°, 48.2°, 54.5°, and 62.8° of TQDs/CC photocatalyst correspond to (101), (004), (200), (105), and (204) of anatase TiO_2_ diffraction planes, respectively, are well-matched to JCPDS data (No. 21-1272) [22,47]. In addition, the grain size of TQDs is calculated to be about 6 nm according to the HR-TEM by using Scherer’s formula, as shown in equation: *D* = 0.90*λ*/*B*cos*θ* [48], where *λ* is the X-ray wavelength, *B* is the full width at half maximum (FWHM) of the most intense peak, *θ* is the Bragg angle, and *D* is crystal size.

The XPS shown in Figure 4c–f aims to characterize the chemical composition of the TQDs/CC photocatalyst. Figure 4c reveals three characteristic elemental peaks of C 1s, O 1s, and Ti 2p in wide-scan XPS spectra [22,49,50]. The high-resolution XPS spectrum of C 1s presents three fitting peaks at 284.8, 286.5, and 288.5 eV (Figure 4b), corresponding to C-C, C-OH, and C-O, respectively [51,52]. The three relevant fitting peaks of the O 1s XPS spectrum at 529.9, 531.4, and 532.8 eV presented Ti-O, C-O-C, and O-O, respectively (Figure 4c) [53]. It should be noted that the peak near 532.8 eV is related to oxygen, which may be related to O_2_ adsorbed on the sample surface. In Figure 4f, two fitting peaks at 458.6 and 464.3 eV are assigned to Ti^4+^ 2p3/2 and Ti^4+^ 2p1/2, respectively, indicating the presence of TiO_2_ [54,55].

### 3.3. Photocatalytic Property of TQDs/CC Photocatalyst

**Figure 5:**
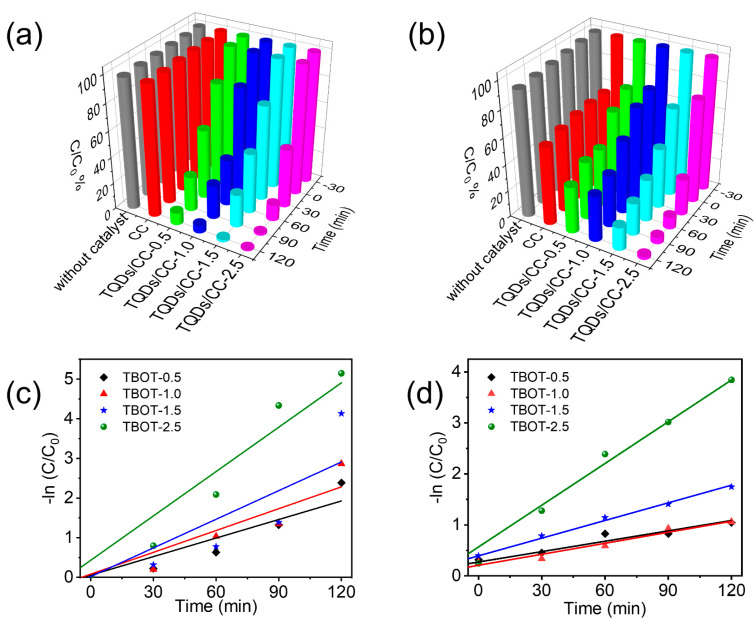
(**a**) Removal rate and pseudo-first-order kinetics curves (**c**) for MO under different TQDs/CC sample photodegradation. (**b**) Remove rate and pseudo-first-order kinetics curves (**d**) for RhB under different TQDs/CC photocatalyst photodegradation.

The photocatalytic behavior of different TQDs/CC photocatalysts was examined using anion MO dye and cationic RhB dye under UV light conditions. Without adding any catalyst, the concentrations of MO and RhB are basically unchanged. Different TQDs/CC photocatalysts were prepared by adding TBOT at 0.5, 1.0, 1.5, and 2.5 g. Figure S3a,b show the absorbance changes in UV-vis absorption spectra of MO and RhB under different TQDs/CC photocatalysis at 0–120 min. UV-vis spectra and corresponding experimental images display MO and RhB gradually fading with increasing illumination time, which demonstrates the TQDs/CC photocatalyst destroyed the chemical structure of dyes [56]. What’s more, the absorbance of dyes decreases gradually with the increase in TBOT after the same exposure time. That is to say, the removal rate of MO and RhB gradually improves with the increase in TBOT (Figure 5a,b), which means that more TBOT leads to the formation of thicker TQD layers to further improve the photocatalytic performance. It is expected that TQDs/CC-2.5 has the best photocatalytic activity for MO and RhB. The removal rate for MO and RhB degraded by TQDs/CC-2.5 is close to 100% after 90 min, indicating that MO and RhB are removed.

Interestingly, Figure 5b portrays that RhB concentration decreased by about 40% in the CC control group before the photodegradation began, while the concentration decrease is slighter in the other groups. The result indicates that CC, rich in hydroxyl and with a porous structure, easily absorbs cationic organic dyes [57]. However, due to TQDs occupying the surface of the CC, the adsorption capacity of the TQDs/CC photocatalyst is inferior to that of CC. Under subsequent light conditions, the as-synthesized photocatalyst rapidly degraded RhB, while the control group remained essentially unchanged. This result suggests that TQDs accelerate the degradation of cationic organic dyes.

To study the kinetics of the photocatalytic removal for MO and RhB by the TQDs/CC photocatalyst, the pseudo-first-order model was used, the equation is −ln(*C*/*C*_0_) = *kt* [58], where *C* and *C*_0_ are concentrations of MO or RhB at times 0 and t, respectively. The *k* value is the pseudo-first-order reaction constant rate. Figure 5c,d reveal pseudo-first-order kinetic fitting linear curves of photocatalytic degradation for MO and RhB by different TQDs/CC. The correlation coefficients and the kinetic constants are shown in Table S1. According to the *k* value, TQDs/CC-2.5 has the highest photocatalytic activity and its degradation rate constants for MO and RhB reach up to 0.0373 and 0.0273 min^−1^, respectively.

### 3.4. Reusability and Stability of TQDs/CC Photocatalyst

**Figure 6:**
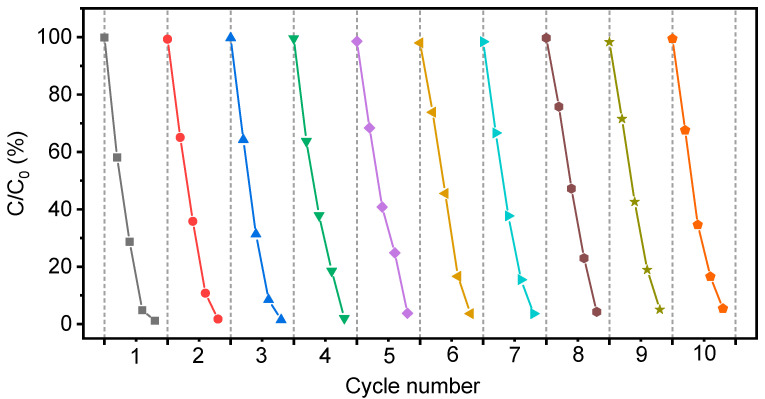
Recycle tests of TQDs/CC-2.5. The cycles of test increases from left to right, and each colored curve represents the number of cycles.

**Figure 7:**
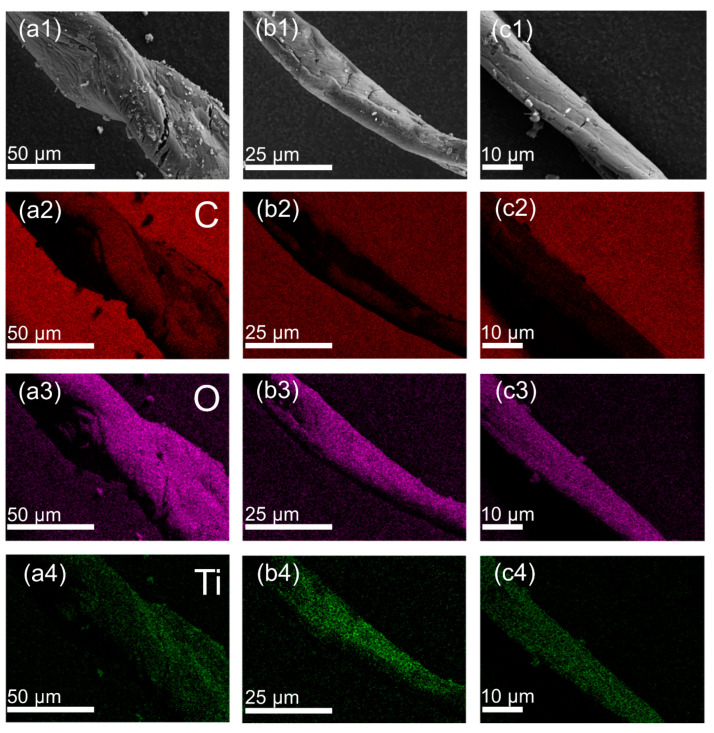
SEM and EDX mapping of TQDs/CC-2.5: (**a1**–**a4**) without photodegradation; (**b1**–**b4**) five recycle tests; (**c1**–**c4**) ten recycle tests.

The reusability and stability of the as-synthesized photocatalyst were tested by degradation for MO using the TQDs/CC-2.5 photocatalyst. The TQDs/CC-2.5 photocatalyst was cleaned with deionized water and dried before each photodegradation experiment. Figure 6 shows the recycle test of the TQDs/CC-2.5 photocatalyst. The result indicates that the photocatalytic efficiency of the TQDs/CC-2.5 photocatalyst is not less than 95% after repeated photodegradation tests for ten cycles, indicating that the TQDs/CC-2.5 has high photocatalytic stability and excellent reusability. As shown in Figure 7(a1,b1,c1), the basic structure of TQDs/CC-2.5 is not damaged after repeated photodegradation for MO above ten cycles. Figure 7(b2–b4) shows C, O, and Ti SEM-EDX mapping results of TQDs/CC-2.5 after recycling tests for five cycles, while Figure 7(c2–c4) corresponds to recycling tests after ten times. Compared to the SEM-EDX mapping of TQDs/CC-2.5 without photocatalytic response (Figure 7(a2–a4)), TQDs firmly remain on the CC surface after five and ten cycles of recycling tests of TQDs/CC-2.5. The photocatalytic activity of the TQDs/CC photocatalyst remained unchanged after more than ten long-term recycling tests, which proved that CC highly stabilized the TQDs. Moreover, it can be inferred that some outermost TQDs are detached from the TQDs/CC during the photodegradation test, and TQDs in the inner further continue to play a role in degrading MO.

### 3.5. Hypothesis of Photodegradation Mechanism

**Figure 8:**
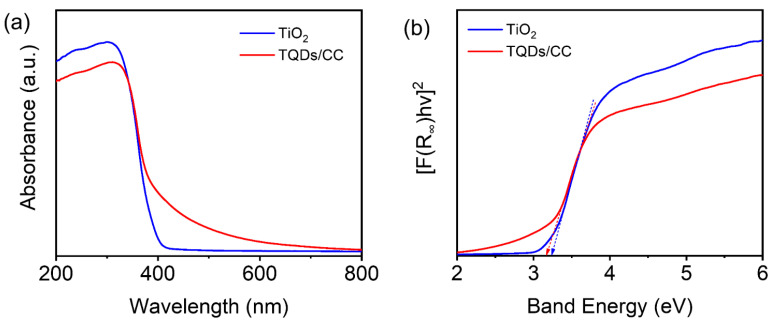
(**a**) UV-vis DRS spectra of TiO_2_ and TQDs/CC-2.5 photocatalyst and (**b**) Kubelka–Munk plots as calculated from the DRS spectra.

To better understand the photodegradation of organic dyes by the TQDs/CC photocatalyst, exploring the mechanism of the photocatalytic activity of the TQDs/CC photocatalyst has been probed. UV-vis DRS spectra were applied to study the optical properties of commercial anatase TiO_2_ and the TQDs/CC-2.5 photocatalyst. The UV-vis DRS spectrum of the TQDs/CC-2.5 photocatalyst gives slight rise to a red shift and the appearance of visible light absorbance in comparison with commercial TiO_2_ in Figure 8a [21]. The band gaps are computed according to the Kubelka–Munk function [59]. As the Kubelka–Munk plots show (Figure 8b), the band gap determined for anatase TiO_2_ is 3.25 eV, while for the TQDs/CC-2.5 photocatalyst it is reduced down to 3.17 eV.

**Figure 9:**
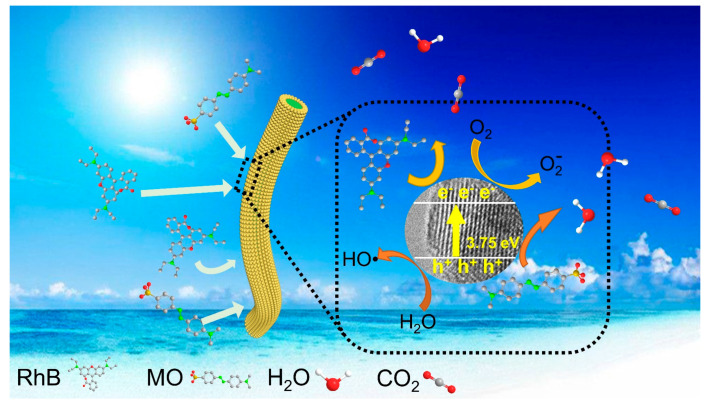
Photocatalytic degradation of MO or RhB by TQDs/CC photocatalyst.

Here, a possible photodegradation mechanism is proposed. Figure 9 shows the catalytic degradation process for MO and RhB by the TQDs/CC photocatalyst. CC containing a large number of hydroxyl groups adsorb organic pollutants from wastewater, like MO or RhB, through coordination or hydrogen bonding [32,57,60]. Meanwhile, the more reactive sites and the narrower band gap of the TQDs/CC photocatalyst compared to commercial TiO_2_ provides the possibility of improving the photocatalytic activity in the visible light region. Under UV light, electrons of TQDs are transferred from the valence band (VB) to the conduction band (CB), generating electron (e^−^) -hole pairs (h^+^) [61]. In the photocatalytic process, O_2_ is reduced to the superoxide radical anion (O_2_^−^) by electrons (e^−^), and the hydroxyl radicals (OH·) formed due to the reaction of the strongly oxidizing photogenerated holes (h^+^) with water [17,62]. O_2_^−^, OH·, or h^+^ further oxidize MO or RhB into small molecules such as H_2_O and CO_2_ without producing toxic and harmful intermediates.

## 4. Conclusions

In summary, the TQDs/CC photocatalyst was prepared with a simple one-step hydrothermal method. As the cotton matrix is a natural biopolymer, the TQDs/CC photocatalyst is eco-friendly and pollution-free while improving the recovery and utilization of TiO_2_. SEM, EDX, TEM, FT-IR, XRD, and XPS were used to verify that the TQDs were decorated on the CC and formed a relatively uniform TQDs layer. Because CC is rich in hydroxyl and has a porous structure, the TQDs/CC photocatalyst has high adsorption and photo-oxidation properties of dyes. The removal rate of anionic MO dye or cationic RhB dye by the best-in-class TQDs/CC-2.5 photocatalyst is still close to 100% after ten cycles of photodegradation, exhibiting an impressive catalyst life. In addition, the structure of TQDs/CC-2.5 is studied by SEM-EDX mapping after ten cycles of photocatalytic stability experiments, which proved that the TQDs/CC photocatalyst has high chemical and physical stability. Such structural characteristics ensure that the loss of TQDs caused by the spalling of TQDs from the CC surface can be avoided when the TQDs/CC photocatalyst is reused. The long-term stability of TQDs/CC in activity and structure directly proves its great application potential in the photodegradation of different types of dyes.

## Data Availability

All data supporting the conclusions of this article are included within the article.

## Supplementary Materials

The following supporting information can be downloaded at: https://www.mdpi.com/article/10.3390/nano12183130/s1, Figure S1: SEM images for CC (**a**) and TQDs/CC sample (**b**), respectively; Figure S2: FT-IR spectrum of commercial anatase TiO_2_; Figure S3: UV-vis curve for MO (**a**) and RhB (**b**) under different TQDs/CC sample photodegradation; Table S1: Correlation coefficient and kinetic constants of MO and RhB photodegradation.
