# Supplementary material for: Natural Cotton Cellulose-Supported TiO2 Quantum Dots for the Highly Efficient Photocatalytic Degradation of Dyes
Source: Nanomaterials (Basel). 2022 Sep 9;12(18):3130. doi: 10.3390/nano12183130 (PMC9504825; doi:10.3390/nano12183130)
Supplement: Supplementary file 1 [file nanomaterials-12-03130-s001.zip › nanomaterials-1871749-supplementary.pdf]

nanomaterials

Article

Natural Cotton Cellulose-Supported TiO_2 Quantum Dots for the Highly Efficient Photocatalytic Degradation of Dyes

Hancheng Shen, Weiwei Zhang, Chunyun Guo, Jing Zhu *, Junjie Cui, Zhonghua Xue * and Peirong Chen *

Department of Applied Chemistry, School of Science, Anhui Agricultural University, Hefei 230036, China
* Correspondence: zhujing@ahau.edu.cn (J.Z.); zhonghua_xue@ahau.edu.cn (Z.X.); chenpeirong@ahau.edu.cn (P.C.)

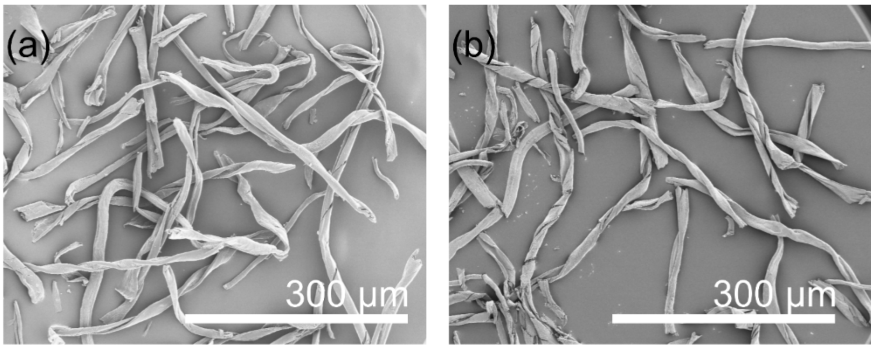

Figure S1. SEM images for CC (**a**) and TQDs/CC sample (**b**), respectively.

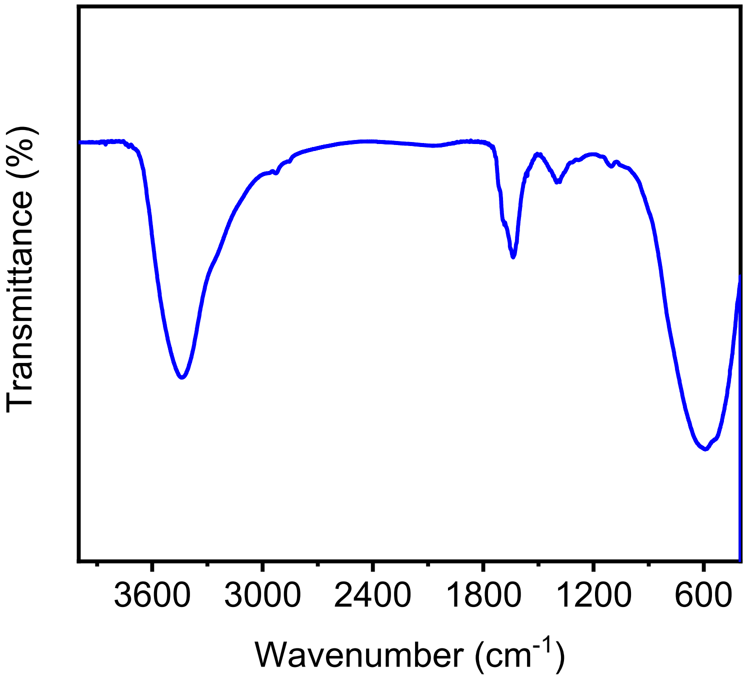

Figure S2. FT-IR spectrum of commercial anatase TiO_2.

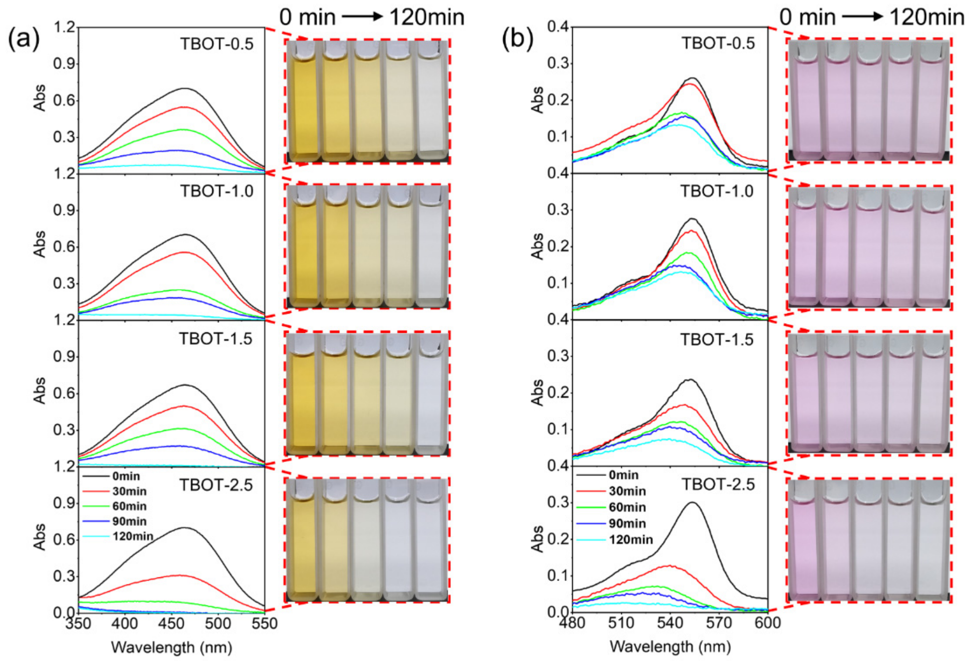

Figure S3. UV-vis curve for MO (**a**) and RhB (**b**) under different TQDs/CC sample photodegradation.

Table S1. Correlation coefficient and kinetic constants of MO and RhB photodegradation.

	MO		RhB	
	**R^2**	**k (min^{-1})**	**R^2**	**k (min^{-1})**
**TQD/CC-0.5**	0.825	0.0156	0.944	0.0068
**TQD/CC-1.0**	0.815	0.0183	0.972	0.0072
**TQD/CC-1.5**	0.670	0.0241	0.994	0.0116
**TQD/CC-2.5**	0.908	0.0373	0.978	0.0273
